# Characterization of Birch Wood Residue after 2-Furaldehyde Obtaining, for Further Integration in Biorefinery Processing

**DOI:** 10.3390/polym13244366

**Published:** 2021-12-13

**Authors:** Maris Puke, Daniela Godina, Mikelis Kirpluks, Prans Brazdausks, Janis Rizikovs

**Affiliations:** 1 Latvian State Institu of Wood Chemistry, Dzerbenes 27, LV-1006 Riga, Latvia; daniela.godina@kki.lv (D.G.); mikelis.kirpluks@kki.lv (M.K.); prans.brazdausks@kki.lv (P.B.); janis.rizikovs@kki.lv (J.R.); 2 Department of Chemsitry, Latvia University of Latvia, Jelgavas 1, LV-1004 Riga, Latvia

**Keywords:** birch wood, pre-treatment, process parameter, lignocellulose, cellulose, 2-furaldehyde

## Abstract

Latvia is a large manufacturer of plywood in Eastern Europe, with an annual production of 250,000 m^3^. In Latvia’s climatic conditions, birch (*Betula pendula*) is the main tree species that is mainly used for plywood production. A significant part of the processed wood makes up residues like veneer shorts, cores, and cut-offs (up to 30%), which have a high potential for value-added products. The aim of this research was to comprehensively characterize lignocellulosic (LC) biomass that was obtained after 2-furaldehyde production in terms of further valorization of this resource. The polymeric cellulose-enriched material can be used in the new biorefinery concept for the production of 2-furaldehyde, acetic acid, cellulose pulp, thermomechanical (TMP) and an alkaline peroxide mechanical (APMP) pulping process. In addition, we experimentally developed the best 2-furaldehyde production conditions to optimize the purity and usability of cellulose in the leftovers of the LC material. The best experimental results in terms of both 2-furaldehyde yield and the purity of residual lignocellulose were obtained if the catalyst concentration was 70%, the catalyst amount was 4 wt.%, the reaction temperature was 175 °C,and the treatment time was 60 min. After process optimization with DesignExpert11, we concluded that the best conditions for maximal glucose content (as cellulose fibers) was a catalyst concentration of 85%, a catalyst amount of 5 wt.%, a temperature of 164 °C, and a treatment time of 52 min.

## 1. Introduction

Nowadays, one of the most important challenges that mankind faces is climate change, which is caused by ever-increasing greenhouse gases (GHG) in the atmosphere produced by the burning and processing of fossil resources. To combat this problem, the European Union (EU) has long pursued a leading role in policies to tackle climate change. In December 2019, the EU made the energy transition one of its main goals and announced that it would pursue a “European Green deal” [1]. The Green Deal can be conceptualized as a roadmap of key policies for the EU’s climate agenda, based on which the Commission has started and will continue to develop legislative proposals and strategies from 2020 onwards. EU climate and energy governance are structured around three main headline targets, concerning: (1) a GHG reduction from 1990 levels, (2) the share of renewable energy in final energy consumption, and (3) an improvement in energy efficiency [2]. To decrease industrial dependence on fossil energy resources, there is an ever-more increasing interest in the integrated biorefinery approach development, in which fossil resources are substituted with renewable, biomass-based resources in a closed-loop system [3]. A renewable and sustainable source of organic feedstock with zero carbon emission is one of the most promising pathways to produce green products and replace fossil resources that are in limited and dwindling supply. 

From biomass-based feedstock, LC biomass is the most commonly available renewable resource [4] that do not compete with food supplies. Moreover, LC biomass is commonly derived in large quantities from forestry and agricultural waste products. Taking this into account, LC biomass has great importance as the main source of biofuels and other high value-added products such as 2-furaldehyde, lactic acid, or monomeric phenols, which can be utilized as building blocks for the production of polymeric materials [5,6]. In addition, LC biomass is a potential source for chemical resource products, such as ethanol, reducing sugars, and 2-furaldehyde, using hydrolysis processing either via an acid-catalyzed or enzymatic route. All pentose-containing material could, in theory, be used as a raw material for 2-furaldehyde production. However, for an economically viable process, 2-furaldehyde industrial production requires a minimum content of around 15% to 20% of pentoses in biomass [7]. Only about one-third of the pentosanes in raw materials can be converted into 2-furaldehyde through the existing production processes. 2-furaldehyde is an important chemical because it is a selective solvent for separating saturated and unsaturated compounds in petroleum refining, gas, oil, diesel fuel and for the high demand of its derivatives, especially 2-furaldehyde alcohol, used mainly in the production of furan resins for foundry sand binders, which is considered the major market for 2-furaldehyde. 

In addition to this, 2-furaldehyde can be used as a starting material to produce a wide range of chemicals, and as such, it is a natural precursor to a range of furan-based chemicals and solvents, including methylfuran, furfuryl alcohol, tetrahydrofurfuryl alcohol, tetrahydrofuran, methyltetrahydrofuran, dihydropyran, and furoic acid. Hydrogenation of the aldehyde group or furan ring remains the most versatile reaction to upgrade furanic components and can be employed to synthesize hydrocarbon fuels directly from furan derivatives. Cleavage of the furan ring by hydrogenolysis can produce alcohols such as 1,5-pentanediol. To synthesize longer-chain hydrocarbons from furfural, adduct formation by aldol condensation and dimerization followed by hydrodeoxygenation can produce C8 to C13 and longer alkanes [8,9,10].

There is no synthetic route available for 2-furaldehyde production in the chemical industry. So far, all of the 2-furaldehyde is exclusively produced by acid hydrolysis and dehydration of pentoses (mainly xylose) contained in LC biomass resources [11,12,13]. In Latvia, the most promising LC feedstock for 2-furaldehyde obtaining is birch chips due to its industrial-scale availability in plywood production as well as its carbohydrate content suitable for effective 2-furaldehyde obtaining [14,15,16]. 

There have been different publications on 2-furaldehyde production by using varying process conditions, such as by using catalyst or starting feedstock, both from our research group as well as others, which focuses on maximizing obtained 2-furaldehyde yields and obtaining various other side-stream products [17,18,19,20,21]. 2-furaldehyde is a useful bio-based chemical that can be used as a starting block for the production of various value-added chemicals, such as solvents, plastics and various additives for the agricultural industry. 2-furaldehyde is only being produced from biological resources, and it has been identified as one of the top 30 most important biomass-based chemicals [22,23]. 

Although 2-furaldehyde obtaining is widely studied and used in industry, for successful process integration into the biorefinery processing chain, it is necessary to effectively utilize all parts of used feedstock to obtain value-added products. Carbohydrates extracted from the glucose-enriched residues obtained from furfural production could be used to produce 5-HMF, levulinic acid, or bioalcohol as well as used as a source of cellulosic fiber. The remaining lignin from this LC residue could be used for manufacturing various platform chemicals, such as aromatics, olefins, dibasic acids, lignin-based epoxydes, and lignin-derived polyurethanes [24,25,26]. 

To be in line with this, more attention is being spent on characterizing LC residue leftover after 2-furaldehyde obtaining. One of the ways for utilizing this residue could be as a pulp and lignin source by using it in various delignification processes such as TMP or sulphate pulping.

The main goal of this paper is to present information regarding obtained LC residue after 2-furaldehyde obtaining, its composition, and to show its potential as a feedstock in various pulping processes. The goal of the study was to obtain 2-furaldehyde at more than 60% of the theoretical amount and the cellulose degradation step at no more than 10%.

## 2. Materials and Methods

### 2.1. Materials and Chemicals

Orthophosphoric acid (H_3_PO_4_) (85%), sulfuric acid (95–97%), D-(+)-cellobiose (≥99%), D-(+)-glucose, (≥95%), D-(+)-xylose (≥99%), L-(+)-arabinose (≥99%), D-(+)-galactose (≥99%), D-(+)-mannose (≥99%), 2-furaldehyde (≥99%), acetic acid (≥99%), 5-hydroxymethylfurfural (5-HMF) (≥99%), levulinic acid (≥98%), and formic acid (≥95%) were purchased from Merck (Darmstadt, Germany) and used without further purification.

### 2.2. Samples

**Figure 1:**
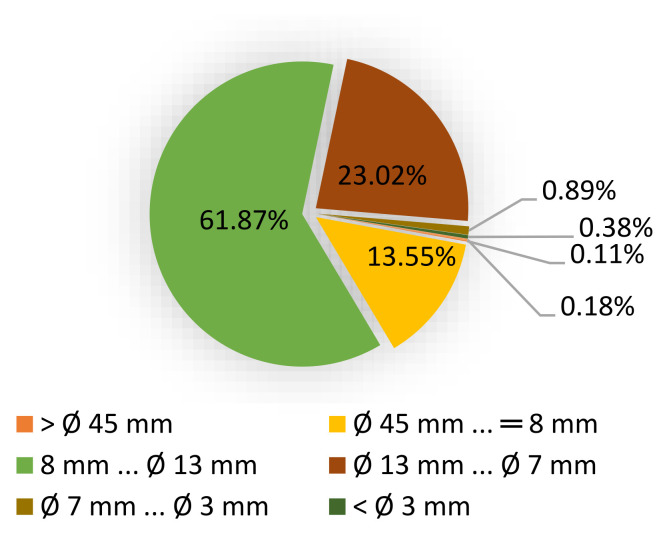
Birch wood chip fractional distribution.

Birch wood chips (BWCs) were supplied by the A/S Latvijas Finieris company “Lignums”, focusing on the production of plywood and processed wood chips. The company supplies BWCs to pulp producers in Scandinavia. We are using standard wood chips, which are used in pulp mills for cellulose production. The fractional distribution is shown in Figure 1.

After obtaining, BWCs were air-dried and stored at 15–20 °C to prevent degradation prior to use. The relative humidity in the laboratory was 25–35%. BWCs were of particle size between 45 and 47 mm.

### 2.3. Catalyzed Pre-Treatment of BWCs

BWCs (particle size 45–47 mm and moisture content W_rel_ = 40.43%) were mixed in a catalyst solution in a blade-type mixer of a special design. Orthophosphoric acid solution of a varied concentration (55–85%) was used as a catalyst. After mixing the chips with a defined amount of the catalyst, the obtained material was treated with a continuous superheated steam flow in an original pilot plant. These value-added products were obtained: condensate containing formic acid, acetic acid, levulinic acid, 5-HMF and 2-furaldehyde, and carbohydrates-enriched LC residue. The diameter of the main reactor camera was 110 mm, its height was 1450 mm, it had a volume of 13.7 L, and a max pressure of 1.2 MPa (bench-scale reactor is shown in our previous publication [21]).

The reactor had two heat insulation systems with automatic equipment to ensure a constant temperature in the reaction zone during the whole process time and with different process parameters. The steam leaving the reactor, which contained mainly a water solution of 2-furaldehyde and acetic acid, was condensed, and samples were taken every 10 min. The steam-treated wood chips lignocellulose (LC) was discharged from the reactor. The chemical composition of the birch chips was determined by the wet chemistry analytical standard methods as described in the Technical Association of the Pulp and Paper Industry (TAPPI) standards [27,28]. All yields of the products and catalyst amounts were calculated on the oven-dried mass of the initial feedstock. For each sample, three parallel experiments were carried out, and the obtained results are shown as the average, with the relative standard deviation (RSD) for all experiments being less than 5%.

### 2.4. Experimental Design

Based on the data described in our previous scientific publication [21] and information available in studies [10,29,30], the following process parameters were set, and after its implementation, it was possible to judge the direction in which to continue the experimental work (Table 1). It is important to note that the experimental design and process parameters depend on the used type of biomass, the catalyst, and the type of hydrolysis reactor. In turn, the constant factors were the moisture of the raw material (w)—40%, and the steam flow rate (v)—120 mL·min^−1^.

In our previous work [21], using the computer program DesignExpert11, the initial full factorial experimental plan was developed, and after its implementation, it was possible to decide in which direction to continue the experimental studies and find the optimal process parameters for the pre-treatment process. Experimental work was performed on the bench equipment for 25 different experiments, continuing the sample listing from our previous experiments [21]. During these twenty-five experiments, in total ninety-four samples with two parallel samples were obtained. Samples were collected in different time increments. All of the condensate samples containing 2-furaldehyde, acetic acid and other compounds were obtained and analyzed by HPLC (Shimadzu 20AD). Furthermore, samples of LC residue were obtained, and their chemical composition was determined. Obtained data are an average value of the 2 parallel experiments of the 94 condensate samples.

### 2.5. HPLC Analysis

The contents of monosaccharides, 2-furaldehyde, 5-HMF, and organic acids in the obtained hydrolysates were determined using a Shimadzu LC-20A HPLC (Shimadzu, Tokyo, Japan) with a refractive index detector. Cellobiose, glucose, xylose, arabinose, galactose, mannose, 2-furaldehyde, acetic acid, 5-HMF, levulinic acid and formic acid (Merck, Darmstadt, Germany) with purity ≥99.0% were used as reference standards. For the cellobiose, glucose, 2-furaldehyde, acetic acid, 5-HMF, levulinic acid and formic acid, we used a Shodex Sugar SH1821 column at 60 °C, with eluent 0.008 M H_2_SO_4_ at a flow rate of 0.6 mL·min^−1^. For the carbohydrate analysis, we used a Shodex Sugar SP0810 column at 80 °C, with deionized water as the mobile phase under a flow rate of 0.6 mL·min^−1^. Samples were neutralized to pH 5–7 with BaCO_3_ and filtered through a 0.2 μm membrane filter before injection. All samples were tested three times.

For each analyzed standard, the equations of the calibration curves are given in our previous publication [21].

## 3. Results and Discussion

### 3.1. Analysis of the Raw Material

The chemical composition of the BWCs was determined, and characteristics are given in Table 2.

The chemical composition of the used material is comparable to information found in the literature [16]. Characteristics of feedstock show that birch chips have the potential to be used in 2-furaldehyde obtaining due to its content of glucose and xylose. Xylose is a raw material for the production of 2-furaldehyde, while glucose-enriched LC residue can be further utilized as a raw material in pulping processes to obtain cellulosic fibers. From the obtained results, it is possible to calculate that the maximum theoretical amount of 2-furaldehyde obtainable from the BWCs is 16.45% on the oven-dried mass (o.d.m.).

### 3.2. Selection of the Initial Pre-Treatment Process Parameters for the Experimental Plan

#### 3.2.1. Condensate Chemical Composition after Hydrolysis

Experimental conditions of these experiments are given in Table 1. After the obtained data (Table 3), it can be seen that the best experimental conditions for 2-furaldehyde and acetic acid production is achieved in experiment No. 18, where the catalyst concentration was 70%, the catalyst amount was 4 wt.%, the reaction temperature was 175 °C, and the treatment time was 60 min. The lowest 2-furaldehyde and acetic acid amount (% of o.d.m.) are for experiment No. 6, where the catalyst concentration was 70%, the catalyst amount was 4 wt.%, the reaction temperature was 155 °C, and the treatment time was 10 min. The total formic acid, levulinic acid, and 5-HMF amount for all of the experiments are below 1%. 

**Figure 2:**
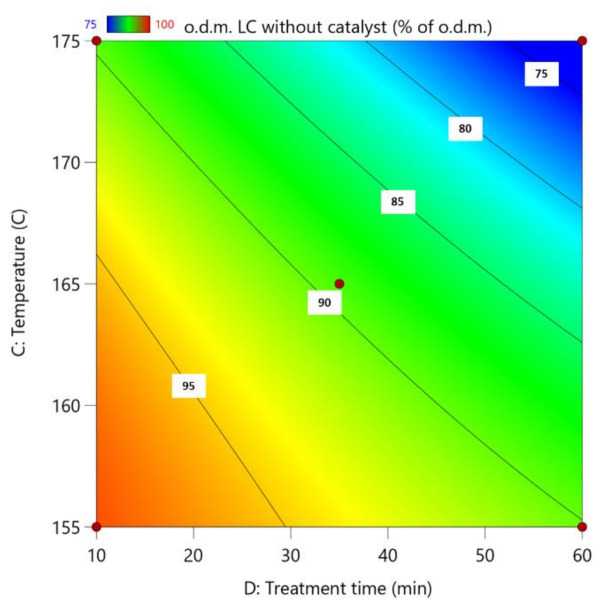
Influence of pre-treatment process parameters on the amount of lignocellulose without catalyst after hydrolysis; the actual factors for the regression model are A = 70 and B = 4.

After Figure 2, it can be observed that the lowest lignocellulose amount was for experiment No. 18, where maximal treatment parameters were used. The highest lignocellulose amount was obtained for experiments No. 15 (catalyst concentration 70%, catalyst amount 3 wt.%, reaction temperature 165 °C and treatment time 10 min) and No. 24 (catalyst concentration 85%, catalyst amount 4 wt.%, reaction temperature 165 °C and treatment time 10 min).

For this regression model, the standard deviation is 1.57 with an R^2^ of 0.9638.

Based on the process results, the following process parameter Equation (1) was obtained:

LC without catalyst = −319.83337 + 0.214028(c) − 2.24448(m) + 5.32509(T) + 1.72771(τ) − 0.006273(c·τ) − 0.009337(T·τ) − 0.017144 (T^2^) (1)

#### 3.2.2. LC Residue Chemical Composition after Hydrolysis

**Figure 3:**
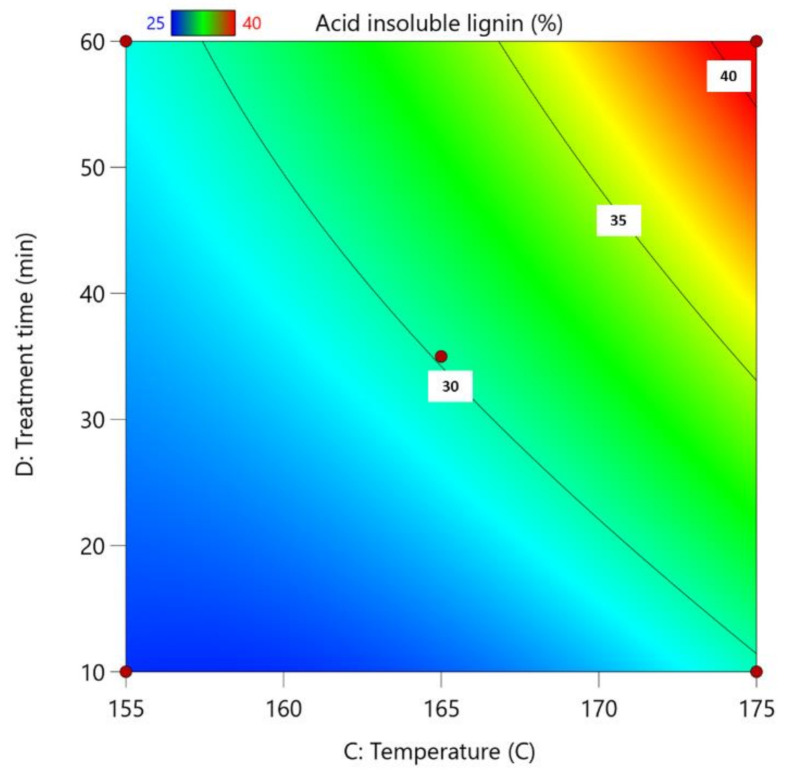
Influence of pre-treatment process parameters on the amount of acid-insoluble lignin in obtained LC residue; the actual factors for the regression model are A = 70 and B = 4.

Experimental conditions of these experiments are given in Table 1. The highest acid-insoluble lignin amount (%) is for experiment No. 18, and the lowest is for experiments No. 6 and No. 19 (Figure 3). The unifying element of these experiments are the catalyst concentration (70%) and the reaction temperature (155 °C). The highest yield of glucan is for experiments No. 3 (catalyst concentration 85%, catalyst amount 4 wt.%, reaction temperature 165 °C and treatment time 60 min) and No. 18 (Table 4). The yields of arabinan for all experiments are below 1%, but the yields of mannan and galactan are below 5%. 

The admixture content was lowest in experiment No. 18, being 1.78 ± 0.02% and 0.30 ± 0.02% for 2-furaldehyde and acetic acid, respectively (Table 5). The conditions for this experiment are as follows: the catalyst concentration was 70%, the catalyst amount was 4 wt.%, the reaction temperature was 175 °C, and the treatment time was 60 min. 

For this regression model, the standard deviation is 0.38 with an R^2^ of 0.994.

Based on the process results, the following process parameter Equation (2) was obtained:

Acid insoluble lignin = 351.58226 + 0.019073(c) + 1.09748(m) − 4.14765(T) − 1.19312(τ) + 0.008137(c·τ) + 0.012927(T^2^) (2)

### 3.3. Changes in the Average Degree of Cellulose Polymerization after the 2-Furaldehyde Obtaining Process

It is very important that the average degree of the polymerization of the cellulose in the LC residue during the 2-furaldehyde obtaining process is decreased to 200. This means that the structure of the wood cell wall is affected, and less energy will be required for the thermomechanical pulping process.

**Figure 4:**
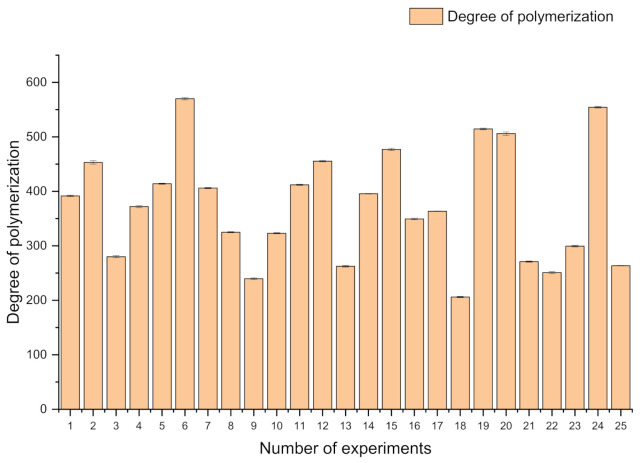
Changes in the degree of cellulose polymerization during the 2-furaldehyde obtaining process.

The changes of the cellulose degree of polymerization in separated LC residuals after the 2-furaldehyde obtaining process indicate the availability of this resource for further processing (Figure 4). For the most promising experiments (No. 18), the degree of polymerization is decreased to 200. This indicates that for this sample, the cell-wall structure is significantly altered, and further pulping of this resource can be implemented at milder conditions, preserving energy resources as well as the quality of the obtained pulp. It could be assumed that a lower degree of polymerization in the obtained LC residuals can further facilitate the pulping process performance, and obtained cellulose fibers in the pulp will be shorter in comparison to the pulping process of the unmodified raw material.

### 3.4. Experimental Design Modulation Using DesignExpert11

To characterize obtained results in terms of process efficiency, results were processed using DesignExpert11. This allows for the obtaining of mathematical equations that predict obtained yields of 2-furaldehyde, acetic acid, and glucose-enriched LC residue by varied input parameters. Obtained parity plots reveal how the predicted results correlate with the obtained actual results, and the obtained surface plots show how process parameters influenced the obtained results. 

Based on the process results, the following process parameter Equation (3) was obtained:

2-furaldehyde = 83.58384 − 0.014482(c) + 0.025299(m) − 1.03242(T) − 0.971688(τ) + 0.000825(c·τ) + 0.014727(m·τ) + 0.005891(T·τ) + 0.003196(T^2^) (3)

**Figure 5:**
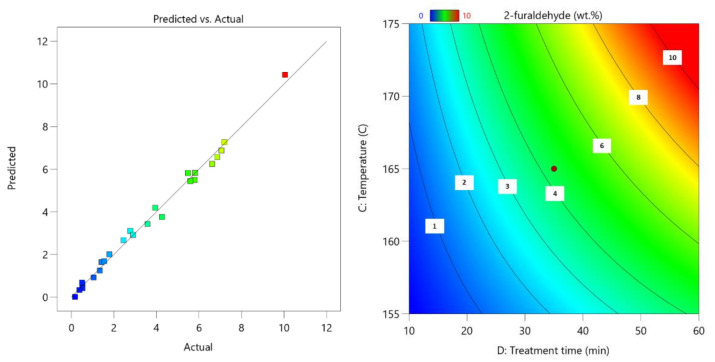
Parity plot of 2-furaldehyde yield (**left**); influence of pre-treatment process parameters on 2-furaldehyde yield (**right**); the actual factors for the regression model are A = 85 and B = 5.

For this regression model, the standard deviation is 0.29 with an R^2^ of 0.993, which indicates that the prepared model correlates closely with the actual obtained data. The surface graph for 2-furaldehyde shows that to obtain the maximum amount of 2-furaldehyde, process conditions have to be optimized using the highest possible temperature as well as the longest process duration, which leads to a higher amount of LC material conversion to 2-furaldehyde (Figure 5). This, of course, does not take into account any unwanted side reactions and degradation that can occur in such harsh conditions. 

Based on the process results, the following process parameter Equation (4) was obtained:

Acetic acid = −19.31076 + 0.093366(c) + 1.82872(m) + 0.064675(T) + 0.173131(τ) − 0.020892(c·m) − 0.001378(τ^2^) (4)

Similar to results obtained for 2-furaldehyde, the acetic acid prediction model corresponds nicely with obtained data with the obtained process equation as shown in Equation (2). 

**Figure 6:**
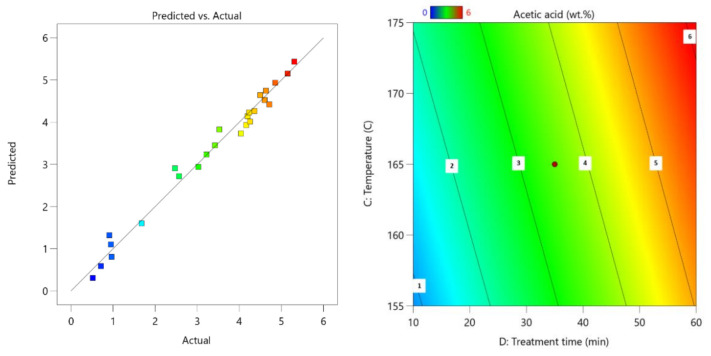
Parity plot of acetic acid (**left**); influence of pre-treatment process parameters on acetic acid yield (**right**); the actual factors for the regression model are A = 85 and B = 5.

For this prediction model, the standard deviation is 0.23 with an R^2^ of 0.9832 (Figure 6).

Next was optimization of the process parameters for maximal glucose content in leftover LC residue after 2-furaldehyde obtaining. Based on the process results, the following process parameter Equation (5) was obtained:

Glucose = 387.26156 + 0.074569(c) + 10.46853(m) + 4.93048(T) + 0.241925(τ) − 0.001571(c·τ) − 0.065291(m·T) − 0.014377(T^2^) − 0.001696(τ^2^) (5)

For this prediction model, the standard deviation is 0.58 with an R^2^ of 0.837.

**Figure 7:**
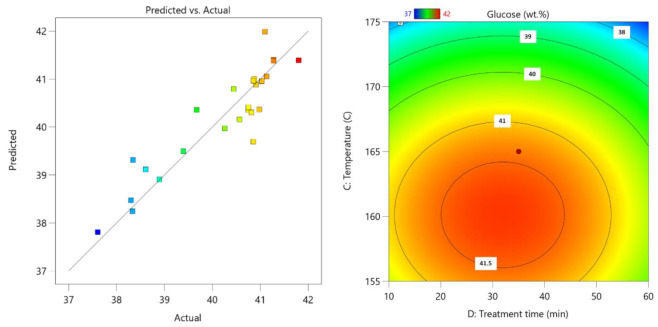
Parity plot of glucose yield (**left**); influence of pre-treatment process parameters on glucose yield (**right**); the actual factors for the regression model are A = 85 and B = 5.

Here, the surface plot reveals that to ensure maximal cellulose fiber conservation in the leftover residual fraction, it is vital that the 2-furaldehyde obtaining temperature is around 162 °C and the process length is around 40 min (Figure 7). Deviation from these conditions leads to either incomplete hemicellulose transformation in milder condition, which leads to a lower relative amount of cellulose, or in the case of a harsher-than-optimal condition, it leads to unnecessary degradation of cellulose fiber in addition to unwanted degradation/condensation reactions, which also lowers the amount of cellulosic fiber in the LC residue. 

### 3.5. Experimental Design Optimization Using DesignExpert11

In DesignExpert11, process parameters were set to obtain the best possible outcome in terms of glucose content in the leftover LC residue (Table 6). Parameter limits were set based on previously obtained results and experimental design [21]. 

**Figure 8:**
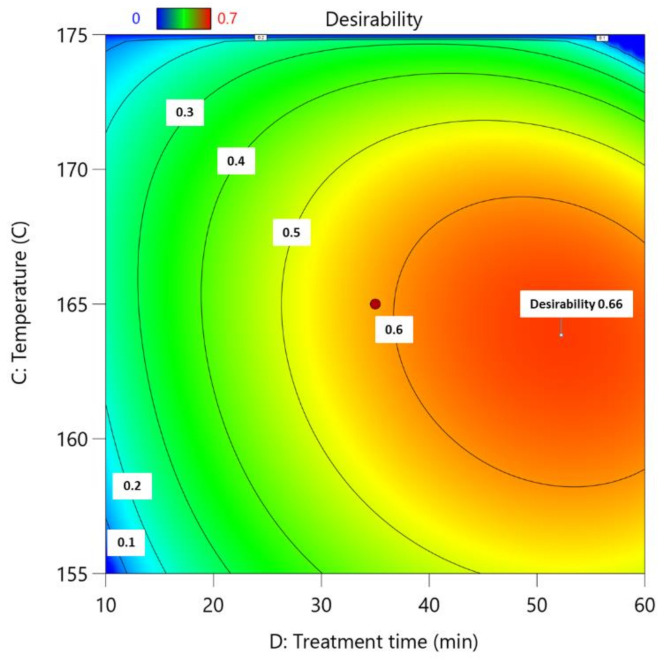
Influence of process time and temperature on total desirability of process; the actual factors for the regression model are A = 85 and B = 5.

Optimized process conditions are shown in Table 7, and the influence of the process time and temperature on the total desirability process is shown in Figure 8. Utilizing these conditions, it is possible to obtain 2-furaldehyde and acetic acid from biomass with yields of 1.6% and 2.29% respectively, while obtaining lignocellulose residue, which is enriched with cellulose. Glucose content in the obtained residue was 41.8%, which is the maximum possible amount based on the starting feedstock. 

## 4. Conclusions

Birch wood chips are a resource that can be successfully integrated into a biorefinery-based processing pathway whilst simultaneously obtaining 2-furaldehyde, acetic acid, and cellulose fiber-enriched LC residue. The best results in terms of both 2-furaldehyde yield and purity of residual lignocellulose were obtained at a catalyst concentration of 70%, a catalyst amount of 4 wt.%, a reaction temperature of 175 °C, and a treatment time of 60 min (No. 18). Obtained residue at these conditions is a promising feedstock for further use in pulping processes to obtain high-quality and purity of cellulosic fibers. After gathering the DesignExpert11 data, the optimized process conditions were as follows: a catalyst concentration of 85%, a catalyst amount of 5 wt.%, a temperature of 164 °C, and a treatment time of 52 min. 

## Figures and Tables

**Table 1:** The process parameters.

No.	Catalyst Conc., (c)	Temperature, (T)	Catalyst Amo., (m)	Treatment Time, (τ)
	%	°C	wt.%	min
1	70	175	4	10
2	70	165	5	10
3	85	165	4	60
4	85	165	3	35
5	85	155	4	35
6	70	155	4	10
7	55	165	3	35
8	70	165	3	60
9	85	175	4	35
10	55	165	5	35
11	70	155	5	35
12	55	155	4	35
13	70	175	3	35
14	70	155	4	60
15	70	165	3	10
16	70	165	4	35
17	85	165	5	35
18	70	175	4	60
19	70	155	3	35
20	55	165	4	10
21	55	175	4	35
22	70	175	5	35
23	70	165	5	60
24	85	165	4	10
25	55	165	4	60

**Table 2:** Chemical composition of BWCs.

Compound	Amount (% of o.d.m.)
Extractives (ethanol-benzene)	4.24 ± 0.06
Extractives (hot water)	1.57 ± 0.44
Glucan	37.84 ± 0.05
Xylan	21.96 ± 0.06
Galactan	0.83 ± 0.05
Arabinan	0.66 ± 0.06
Mannan	1.56 ± 0.50
Acid-insoluble lignin	19.42 ± 0.04
Acid-soluble lignin	3.71 ± 0.06
Ash	0.60 ± 0.01
Acetyl group amount	4.80 ± 0.30
Other unidentified compounds	1.32 ± 0.05

**Table 3:** The chemical composition of condensate after hydrolysis.

No. of Experiments	Amount, %.o.d.m.				
	Formic Acid	Acetic Acid	Levulinic Acid	5-HMF	2-Furaldehyde
1	0.22 ± 0.01	1.68 ± 0.01	0.05 ± 0.04	<0.01	1.42 ± 0.10
2	0.14 ± 0.02	0.91 ± 0.03	<0.01	<0.01	0.51 ± 0.04
3	0.59 ± 0.02	4.86 ± 0.01	0.05 ± 0.01	0.03 ± 0.01	7.05 ± 0.11
4	0.35 ± 0.02	3.53 ± 0.01	0.04 ± 0.01	<0.01	2.78 ± 0.04
5	0.29 ± 0.11	3.22 ± 0.03	0.03 ± 0.01	<0.01	1.55 ± 0.19
6	0.09 ± 0.05	0.52 ± 0.04	<0.01	<0.01	0.17 ± 0.09
7	0.38 ± 0.03	2.47 ± 0.11	0.04 ± 0.01	<0.01	2.45 ± 0.07
8	0.52 ± 0.01	4.71 ± 0.01	0.05 ± 0.01	0.02 ± 0.01	5.60 ± 0.02
9	0.53 ± 0.06	4.60 ± 0.01	0.05 ± 0.01	0.02 ± 0.01	6.62 ± 0.09
10	0.40 ± 0.01	4.36 ± 0.02	0.03 ± 0.01	0.01 ± 0.01	4.26 ± 0.02
11	0.31 ± 0.04	3.42 ± 0.02	0.03 ± 0.01	<0.01	1.78 ± 0.03
12	0.28 ± 0.11	3.03 ± 0.03	0.03 ± 0.01	<0.01	1.34 ± 0.04
13	0.48 ± 0.06	4.25 ± 0.02	0.06 ± 0.01	0.02 ± 0.01	5.79 ± 0.05
14	0.40 ± 0.01	4.20 ± 0.01	0.04 ± 0.01	0.01 ± 0.01	2.91 ± 0.20
15	0.13 ± 0.03	0.71 ± 0.03	0.01 ± 0.01	<0.01	0.38 ± 0.01
16	0.40 ± 0.07	4.04 ± 0.01	0.03 ± 0.01	<0.01	3.58 ± 0.01
17	0.43 ± 0.01	4.16 ± 0.01	0.04 ± 0.01	0.01 ± 0.01	3.94 ± 0.03
18	0.67 ± 0.04	5.3 ± 0.02	0.08 ± 0.02	0.06 ± 0.01	10.04 ± 0.02
19	0.27 ± 0.07	2.57 ± 0.01	0.03 ± 0.01	<0.01	1.05 ± 0.14
20	0.15 ± 0.07	0.96 ± 0.01	<0.01	<0.01	0.52 ± 0.08
21	0.47 ± 0.03	4.24 ± 0.01	0.05 ± 0.01	0.02 ± 0.01	5.48 ± 0.04
22	0.54 ± 0.03	4.63 ± 0.01	0.05 ± 0.01	0.02 ± 0.01	6.85 ± 0.04
23	0.62 ± 0.01	5.15 ± 0.01	0.05 ± 0.02	0.03 ± 0.01	7.19 ± 0.01
24	0.15 ± 0.04	0.95 ± 0.01	0.02 ± 0.01	<0.01	0.52 ± 0.14
25	0.52 ± 0.08	4.50 ± 0.02	0.02 ± 0.01	0.02 ± 0.01	5.82 ± 0.03

**Table 4:** The yield of carbohydrates in lignocellulose after hydrolysis.

No. of Experiments	Amount, %.o.d.m.				
	Glucan	Xylan	Arabinan	Galactan	Mannan
	40.10 ± 4.00	21.90 ± 0.51	0.51 ± 0.21	2.09 ± 0.17	1.14 ± 0.08
1	40.12 ± 5.11	22.31 ± 0.30	0.82 ± 0.54	1.87 ± 0.12	1.30 ± 0.16
2	46.12 ± 6.00	15.51 ± 1.71	0.42 ± 0.23	1.86 ± 0.18	0.96 ± 0.05
3	40.13 ± 6.12	20.63 ± 0.20	0.51 ± 0.31	1.90 ± 0.12	1.06 ± 0.01
4	39.00 ± 6.00	21.71 ± 0.21	0.53 ± 0.22	1.79 ± 0.15	0.88 ± 0.05
5	36.12 ± 5.10	22.81 ± 0.08	0.41 ± 0.20	1.86 ± 0.32	0.99 ± 0.04
6	41.10 ± 5.11	21.60 ± 0.50	0.31 ± 0.10	2.26 ± 0.19	0.74 ± 0.03
7	43.13 ± 5.10	17.20 ± 0.40	0.27 ± 0.10	2.32 ± 0.14	0.76 ± 0.05
8	44.12 ± 5.00	13.51 ± 0.51	0.29 ± 0.11	1.75 ± 0.01	0.59 ± 0.04
9	42.11 ± 5.10	19.86 ± 0.04	0.38 ± 0.11	2.35 ± 0.03	0.80 ± 0.04
10	39.11 ± 4.13	22.14 ± 0.06	0.37 ± 0.11	2.08 ± 0.02	0.99 ± 0.05
11	38.00 ± 4.10	22.11 ± 0.06	0.47 ± 0.03	2.37 ± 0.01	0.97 ± 0.05
12	45.11 ± 5.12	14.21 ± 0.41	0.30 ± 0.20	0.74 ± 0.19	0.97 ± 0.24
13	40.11 ± 5.00	19.12 ± 0.71	0.60 ± 0.40	0.69 ± 0.10	1.11 ± 0.12
14	37.13 ± 5.00	22.50 ± 0.06	0.61 ± 0.22	0.84 ± 0.01	1.08 ± 0.04
15	42.14 ± 6.00	18.83 ± 0.24	0.38 ± 0.05	0.82 ± 0.07	0.72 ± 0.02
16	43.10 ± 6.11	18.47 ± 0.11	0.38 ± 0.10	0.56 ± 0.13	0.72 ± 0.08
17	46.10 ± 4.12	17.82 ± 0.10	0.17 ± 0.11	0.90 ± 0.01	0.80 ± 0.03
18	39.10 ± 3.13	22.86 ± 0.14	0.29 ± 0.07	2.60 ± 0.04	0.71 ± 0.05
19	37.11 ± 4.00	23.21 ± 0.51	0.33 ± 0.07	2.69 ± 0.02	0.83 ± 0.08
20	43.13 ± 5.00	15.20 ± 0.11	0.22 ± 0.03	1.98 ± 0.01	0.69 ± 0.05
21	45.12 ± 6.13	12.91 ± 0.21	0.22 ± 0.06	1.82 ± 0.18	0.53 ± 0.02
22	44.12 ± 5.10	16.43 ± 0.43	0.23 ± 0.03	1.59 ± 0.08	0.56 ± 0.03
23	37.00 ± 4.00	22.66 ± 0.02	0.37 ± 0.06	3.08 ± 0.02	0.84 ± 0.05
24	44.12 ± 3.00	15.21 ± 0.71	0.26 ± 0.02	2.45 ± 0.14	0.67 ± 0.01

**Table 5:** The yield of admixtures in condensate left in lignocellulose residue after hydrolysis.

No. of Experiments	Amount, %.o.d.m.				
	Formic Acid	Acetic Acid	Levulinic Acid	5-HMF	2-Furaldehyde
	0.53 ± 0.01	1.26 ± 0.01	0.70 ± 0.04	0.40 ± 0.01	3.03 ± 0.10
1	0.44 ± 0.02	1.87 ± 0.03	0.63 ± 0.03	0.37 ± 0.01	3.02 ± 0.04
2	0.51 ± 0.02	0.46 ± 0.01	0.82 ± 0.03	0.43 ± 0.05	2.31 ± 0.11
3	0.51 ± 0.02	1.10 ± 0.01	0.63 ± 0.02	0.36 ± 0.01	2.87 ± 0.04
4	0.46 ± 0.11	1.22 ± 0.03	0.55 ± 0.04	0.35 ± 0.01	2.82 ± 0.19
5	0.40 ± 0.05	2.58 ± 0.04	0.48 ± 0.01	0.34 ± 0.02	2.93 ± 0.09
6	0.37 ± 0.03	1.07 ± 0.11	0.46 ± 0.12	0.40 ± 0.01	2.68 ± 0.07
7	0.35 ± 0.01	0.59 ± 0.01	0.62 ± 0.01	0.39 ± 0.01	2.39 ± 0.02
8	0.48 ± 0.06	0.42 ± 0.01	0.77 ± 0.04	0.42 ± 0.01	2.17 ± 0.09
9	0.44 ± 0.01	0.69 ± 0.02	0.62 ± 0.02	0.39 ± 0.01	2.72 ± 0.02
10	0.39 ± 0.04	1.12 ± 0.02	0.53 ± 0.04	0.37 ± 0.01	2.72 ± 0.03
11	0.43 ± 0.11	1.40 ± 0.03	0.50 ± 0.03	0.37 ± 0.01	2.93 ± 0.04
12	0.50 ± 0.06	0.57 ± 0.02	0.74 ± 0.02	0.40 ± 0.01	2.33 ± 0.05
13	0.39 ± 0.01	0.84 ± 0.01	0.54 ± 0.01	0.42 ± 0.01	2.61 ± 0.21
14	0.43 ± 0.03	2.52 ± 0.03	0.49 ± 0.04	0.40 ± 0.01	3.06 ± 0.01
15	0.41 ± 0.07	0.71 ± 0.01	0.61 ± 0.03	0.42 ± 0.01	2.83 ± 0.01
16	0.43 ± 0.01	0.72 ± 0.01	0.66 ± 0.06	0.43 ± 0.01	2.82 ± 0.03
17	0.56 ± 0.04	0.30 ± 0.02	1.09 ± 0.06	0.44 ± 0.03	1.78 ± 0.02
18	0.51 ± 0.07	1.65 ± 0.01	0.60 ± 0.02	0.37 ± 0.01	2.77 ± 0.14
19	0.45 ± 0.07	2.06 ± 0.01	0.58 ± 0.01	0.37 ± 0.01	3.18 ± 0.08
20	0.63 ± 0.03	0.57 ± 0.01	0.84 ± 0.01	0.43 ± 0.01	2.38 ± 0.04
21	0.71 ± 0.03	0.44 ± 0.01	0.92 ± 0.04	0.42 ± 0.01	2.22 ± 0.04
22	0.68 ± 0.01	0.48 ± 0.01	0.84 ± 0.01	0.43 ± 0.01	2.28 ± 0.01
23	0.50 ± 0.04	2.05 ± 0.01	0.62 ± 0.04	0.40 ± 0.03	3.15 ± 0.14
24	0.51 ± 0.08	0.44 ± 0.01	0.68 ± 0.04	0.48 ± 0.01	2.05 ± 0.03

**Table 6:** Set parameters and their limits for experimental model optimization.

Name	Goal	Lower Limit	Upper Limit	Lower Weight	Upper Weight	Importance
A:Catalyst conc.	Is in range	55	85	1	1	1
B:Catalyst amo.	Is in range	3	5	1	1	1
C:Temperature	minimize	155	175	1	1	1
D:Treatment time	is in range	10	60	1	1	5
2-furaldehyde	maximize	0	11	1	1	5
Acetic acid	none	0.52	5.31	1	1	3
Glucose	maximize	37.61	41.80	1	1	5
o.d.m. LC without catalyst	none	75.21	98.83	1	1	3
Acid insoluble lignin	none	25.50	41.71	1	1	3

**Table 7:** Obtained optimal process conditions.

No.	Catalyst Conc. (c), %	Catalyst Amo. (m), wt.%	Temperature (T), °C	Treatment Time (τ), min	2-Furaldehyde	Glucose	Desirability
1	85	5	164	52	6	41	0.7

## Data Availability

Not applicable.
